# Diet, Gut Microbiota and Non-Alcoholic Fatty Liver Disease: Three Parts of the Same Axis

**DOI:** 10.3390/cells9010176

**Published:** 2020-01-10

**Authors:** Sergio Quesada-Vázquez, Gerard Aragonès, Josep M Del Bas, Xavier Escoté

**Affiliations:** 1 Unitat de Nutrició i Salut, Centre Tecnològic de Catalunya, Eurecat, 43204 Reus, Spain; sergio.quesada@eurecat.org (S.Q.-V.); josep.delbas@eurecat.org (J.M.D.B.); 2 Department of Biochemistry and Biotechnology, Universitat Rovira i Virgili, Nutrigenomics Research Group, 43007 Tarragona, Spain; gerard.aragones@urv.cat

**Keywords:** Non-Alcohol Fatty Liver Disease, gut microbiota, NAFLD, dysbiosis, bacterial translocation

## Abstract

Non-Alcoholic Fatty Liver Disease (NAFLD) is the most common liver disease in the world. NAFLD is principally characterized by an excessive fat accumulation in the hepatocytes. Diet is considered as one of the main drivers to modulate the composition of gut microbiota, which participate in different processes, affecting human metabolism. A disruption in the homeostasis of gut microbiota may lead to dysbiosis, which is commonly reflected by a reduction of the beneficial species and an increment in pathogenic microbiota. Gut and liver are in close relation due to the anatomical and functional interactions led by the portal vein, thus altered intestinal microbiota might affect liver functions, promoting inflammation, insulin resistance and steatosis, which is translated into NAFLD. This review will highlight the association between diet, gut microbiota and liver, and how this axis may promote the development of NAFLD progression, discussing potential mechanisms and alterations due to the dysbiosis of gut microbiota. Finally, it will revise the variations in gut microbiota composition in NAFLD, and it will focus in specific species, which directly affect NAFLD progression.

## 1. Introduction

Non-Alcohol Fatty Liver Disease (NAFLD) is the most common liver disease in the world [1]. An incidence between 20% and 30% is estimated within of the adult population in Western countries. Meanwhile, in Eastern societies, this disease presents a lower prevalence, although some recent studies are pointing at the fact that its incidence is rising due to changes in Eastern nutritional habits, together with a decreasing of physical activity that is typical of a sedentary lifestyle (“Westernized society”) [2]. Regarding nutrition, an improper and excessive intake of saturated fats and caloric oversupply, together with a low intake of vegetables, fruits, proteins, grains and ω3-fatty acids, are key causes to develop NAFLD [3].

NAFLD is principally characterized by an excessive fat accumulation in the hepatocytes (hepatic steatosis). Although NALFD shares many characteristic features with alcoholic liver disease (ALD), NALFD is not induced by the consumption of toxic levels of alcohol [4]. The excessive fat accumulation in the liver is strongly associated with multifactorial risk factors such as obesity, leptin and insulin resistance (IR), dyslipidemia and metabolic syndrome. However, these processes and the causality of these factors are not fully understood [5,6].

NAFLD shows two main stages: The most common stage is nonalcoholic fatty liver (NAFL), a non-progressive and less-dangerous state of the liver condition, whereas nonalcoholic steatohepatitis (NASH) is less frequent, but its potential progression to advanced liver damage is worryingly difficult to revert, and could trigger worst diagnoses such as fibrosis progression, cirrhosis or even hepatocarcinoma (HCC) [2,7]. The principal causes of NASH are steatosis, hepatocyte ballooning and lobular inflammation, and it can only be diagnosed by liver biopsy, which is the only existing reliable diagnosis method nowadays [7]. Several studies have also described age and obesity as risk factors to develop NAFLD, but there are other less known factors that need to be further studied, such as the role of gut microbiota in the NAFLD progression.

Gut microbiota is a complex and dynamic community of different microorganisms which are in symbiotic relationship with the host, exerting a marked influence on several aspects of the host metabolism, maintaining immune health, participating in the metabolic homeostasis and protecting the host against pathogenic infection [8]. Diet is considered as one of the main drivers to modulate the composition of gut microbiota in terms of species richness. Dysbiosis, which is defined as any alteration that affects gut bacterial composition, and is commonly reflected by a reduction of the species number, has been associated with the pathogenesis of several inflammatory diseases and potential infections [9]. These studies foster a better understanding of interindividual species’ variations, the heterogeneity of bacterial communities along and across the intestinal tract, functional redundancy and the need to distinguish the cause from the effect in states of dysbiosis. Mainly, six different phyla are present in the gut microbiota: *Firmicutes, Bacteroidetes, Proteobacteria, Verrucomicrobia, Actinobacteria* and *Fusobacteria* [9]. As previously mentioned, these bacteria might participate in different important processes that affect the human metabolism, including the fermentation of diet polysaccharides, the regulation of bile acid production, the contribution to regulate the choline metabolism and the processes of energy harvest, providing protection against pathogens or even stimulating the endogenous ethanol production [10,11]. Thus, microbiota contribute to the whole intestinal homeostasis. Despite the numerous beneficial aspects of the gut microbiota over host homeostasis, sometimes an excessive proliferation of particular species may be translated into an overproduction of some metabolites that may exert a harmful effect for the intestine and even provoke a systemic inflammation in the worst scenario [12].

Gut and liver are in close relation due to anatomical and functional interactions led by the portal vein [13]. Indeed, the portal vein supplies 70% of the total amount of blood in the liver, thereby the liver is exposed to factors that are mostly originated from the gut. These factors are nutrients and metabolites needed for a proper homeostasis. In other cases, the liver can receive other products directly originated by the gut microbiota, such as endotoxins, peptidoglycans and even complete bacteria, which may cause a large deregulation of several metabolic pathways presented in the liver [14]. This constant influx of microbial-derived products from the intestine to the liver generate a response from pathogen-recognition-receptors located at plasmatic membrane of several hepatic cells, such as the Kupffer cells (stellate macrophages), sinusoidal cells, biliary epithelial cells and hepatocytes [14]. Numerous studies have demonstrated that altered intestinal microbiota might affect in some way the liver functions, causing inflammation, insulin resistance (IR), and fat accumulation, which is translated into obesity and NAFLD as well [15]. In the present study, we have reviewed the latest studies to categorize the effects of intestinal dysbiosis, the role of the diet in this disruption, and the identification of specific gut bacteria mainly associated with NAFLD progression.

## 2. Effects of Dysbiosis in the Gut Microbiota

Gut microbiota is a highly dynamic entity and presents a constant flow in its composition. These variations in the percentages of different bacterial species depend upon several environmental factors with different impacts in the gut microbiota composition. Among others, these environmental factors include the intestinal mucosa state (which directly affects the degree of permeability of the gut barrier), the immune system health of the host (which promotes an increased proliferation of particular and hazardous species in case of immune deficits), drugs presence (because some bacteria are more sensitive to particular medicines which allow proliferation of other species to occupy the empty niche), the type of diet (food rich in fats, fiber or some phytochemicals directly affects the proliferation of specific bacteria) and even other microbiota members [9]. Therefore, these environmental factors might produce stressful culture conditions that can alter the natural composition of the gut microbiota by decreasing microbial diversity, known as dysbiosis, and they may be the cause of increased risk to develop some diseases [16]. Indeed, dysbiosis is directly related with an increased intestinal permeability as a consequence of some aspects, including the epithelial barrier deterioration, small intestinal bacterial overgrowth, tight junctions’ alteration, and even the whole bacterial translocation, causing endotoxemia, which might reach and damage the liver through the portal vein [11,17,18,19].

### 2.1. Obesity and HF Diets Lead to Gut Microbiota Dysbiosis

Microbiota plays a role in obesity development, and that was confirmed in different studies [20]. Food oversupply, food shortage or even changes on food composition are facts that may contribute to a dysbiosis state [21]. Indeed, preclinical studies using different mice models have demonstrated that both obesity and a specific diet that drives to obesity (known as a high fat diet (HFD), characterized by a higher percentage of energy in the form of saturated lipids), induce gut microbiota dysbiosis and an overgrowth of some bacteria phyla and a reduction of other phyla, which cause undesired consequences, such as intestinal inflammation or epithelial barrier disruption [22]. Moreover, in some HFD models, it has also been suggested that gastrointestinal microbiome alterations may affect NAFLD pathogenesis by enhancing its development through different pathways, including an increase of energy harvesting, a rise in metabolism harvesting and an increase in the expression levels of some pro-inflammatory cytokines in liver cells [23]. Additionally, some studies have demonstrated differences in the gut bacterial composition between healthy patients and patients with obesity-related NAFLD, which may suggest a connection between gut microbiota dysbiosis and obesity-related NAFLD progression [24].

An archetypal Western diet is generally characterized by high intakes of red and processed meats, pre-packaged foods, butter and fried foods, high-fat dairy products, eggs and high-sugar drinks. Thus, a Western diet is not only associated with a higher susceptibility to develop obesity, but also to several metabolic disarrangements, some of which are regulated through the signaling pathway of the Toll-like receptor (TLR) family, which is activated by lipopolysaccharide (LPS, a component of the outer membrane gram-negative bacteria) [25].

### 2.2. Role of LPS in Gut Microbiota Dysbiosis

When LPS levels increase in the human systemic circulation, it may indicate a potential dysbiosis of the gut microbiota [26]. NAFLD and obesity are associated with higher gut barrier permeability, causing metabolic endotoxemia and an increase in the blood levels of LPS. Subsequently, it also causes hepatic and systemic inflammation as well as alterations in gene expression, hormone secretion, and energy consumption in distal peripheral tissues, such as the white adipose tissue [27]. This LPS role was corroborated in a preclinical study in which mice injected with LPS showed a similar phenotype than those obtained after a high fat diet (body weight gain, IR and increased NAFLD progression) [15]. Besides, *in vitro* studies have demonstrated that fatty acids can promote LPS absorption, presumably by inducing endoplasmic reticulum stress in epithelial cells and inhibiting the formation of tight junctions between these cells [13].

### 2.3. Role of Short-Chain Fatty Acids (SCFAs) in Gut Microbiota Dysbiosis

Another metabolic disarrangement, and a consequence of the Western diet, is an altered pattern of the short-chain fatty acid (SCFA) production [25]. SCFAs are mainly derived from intestinal microbial fermentation of indigestible food when dietary fibers are fermented in the colon [28], and afterwards, these SCFAs can be absorbed by the intestine [29]. Acetate, propionate and butyrate are the three most common SCFAs [30], and during lipid digestion, SCFAs are primarily absorbed and reach the liver through the portal vein, where acetate can be used as an energy source [31,32,33].

Saturated fatty acid (SFA) can also promote a mitochondrial dysfunction because of changes in the endoplasmic reticulum due to oxidative stress, impaired phospholipid metabolism and raised IR. These affections could induce hepatic steatosis, promoting liver inflammation via an enhancement of LPS-induced inflammasomes in hepatocytes [34,35].

### 2.4. Effect of Choline Deficiency in Gut Microbiota Dysbiosis

In the liver, lipids from the diet, together with apolipoproteins, cholesterol, cholesteryl esters and triglycerides, are assembled to form the nascent very-low-density lipoprotein (VLDL) [32]. VLDL production and the hepatic lipid transfer are modulated by dietary choline, and the choline metabolism is affected by the gut microbiota dysbiosis [33]. In fact, choline-depleted diets are applied in animal models to induce NAFLD, and this liver alteration is reflected into lowered amounts of VLDL and a reduced lipid β-oxidation [33]. Consequently, choline depletion causes cholesterol metabolism disorders, alterations in the c-ytokines pattern, increased hepatocyte oxidative stress and a higher deposition of fatty acids, which triggers inflammation, lipotoxicity and fibrosis [33]. In parallel, choline deficiency is modulated by the conversion of choline to trimethylamine (TMA) [34]. It has been demonstrated that gut bacteria are essential to transform the dietary choline into TMA [31]. TMA can be absorbed and metabolized in the liver into trimethylamine N-oxide (TMAO) [35], which is a metabolite that seems to be involved in the development of metabolic diseases acting as a link between inflammation and obesity [36]. TMAO affects the liver through modulating glucose metabolism, causing inflammation in the adipose tissue and influencing lipid absorption and cholesterol homeostasis [37].

### 2.5. Role of Bile Acids in Gut Microbiota Dysbiosis

Bile acids are amphipathic molecules synthesized in hepatocytes as primary bile acids (cholic acid and chenodeoxycholic acid), both secreted in the lumen of the duodenum, where the bacterial flora transform them into the secondary bile acids (deoxycholic acid and lithocholic acid) [35]. All four of these bile acids can be reabsorbed into the gut and returned to the liver in a process known as enterohepatic circulation [35]. One of the main physiologic functions of bile acids is to emulate fats and bring them near the intestinal brush border membrane, which results in fat absorption in the gut [36]. Besides, HFD modifies the bile acid composition, which might influence the environment in the gut and cause changes in the intestinal conditions, which are more susceptible to induce a dysbiosis state [37]. Moreover, bile acids have other metabolic actions in the body resembling those of hormones, acting through the specific farnesoid X receptor (FXR or bile acid receptor (BAR)), which develops a key role in the control of hepatic de novo lipogenesis, VLDL and plasma triglyceride turnover [38,39]. In fact, a study demonstrated that gut microbiota is able to modify bile acid secretion through FXR stimulation, thereby fostering lipid peroxidation and hepatic steatosis [40]. Finally, bile acids have bacteriostatic and antimicrobial properties that foster a reduction of the microbiota found in the small intestine and biliary tract [41]. Therefore, dysbiosis may induce changes in the bile acids’ production and intestine reabsorption, and at the same time, a reduction in the bile acids’ secretion allows the proliferation of a particular bacterial species, becoming a vicious circle of microbiota dysbiosis.

### 2.6. Effect of Endogenous Alcohol in Gut Microbiota Dysbiosis

Other processes that can be affected by the gut microbiota dysbiosis is the endogenous ethyl alcohol production through the fermentation of carbohydrates in the intestinal lumen [42]. Endogenous ethyl alcohol reaches the liver by the portal vein, which contributes to induce the liver damage that aggravates the pathology of NAFLD [42]. Indeed, there was ethyl alcohol detected which was exhaled in the breath in obese mice, although these animals were not fed with any alcohol [43]. Besides, clinical studies have demonstrated that NAFLD patients presented higher levels of blood ethanol concentrations (BACs) than healthy patients, suggesting that the worsening liver damage was contributed by endogenous ethanol production [42,43,44].

Thus, to deeper understand the processes that conducts to intestinal dysbiosis, it is necessary to identify the factors and mechanisms which originate the changes of gut microbiota in order to design accurate strategies aimed to prevent and to treat intestinal dysbiosis.

## 3. Role of Diet in NAFLD Progression and Gut Microbiota Dysbiosis

As commented in previous sections, NAFLD is strongly related with obesity [41]. As expected, in the NAFLD patients, mRNA expression of fatty acid synthase (FAS, a key enzyme in the hepatic de novo lipogenesis) increased, together with the LPS receptors TLR2 and TLR4. Consequently, the circulating levels of gut microbiota-derived metabolites were analyzed observing that TMAO, glycocholic acid and deoxycholic acid plasma levels were significantly increased in NAFLD patients, suggesting the use of circulating microbiota-derived metabolites as a scoring system for the clinical diagnosis of NAFLD [45]. The up-regulation of lipogenic genes due to a high intake of saturated fatty acids (SFAs) triggers triglyceride (TG) formation [46]. TGs from dietary fats are metabolized to diglycerols, monoacylglycerols and fatty acids in the intestinal lumen and transported by enterocytes to the portal vein, where finally fatty acids are introduced in hepatocytes [47]. These fatty acids might be saturated or unsaturated (monounsaturated fatty acids, MUFAs, or polyunsaturated fatty acids, PUFAs) with a different impact upon hepatic health. Indeed, it is described that the concentration of MUFAs is increased in NAFLD patients compared to healthy controls [48]. In contrast, lower circulating levels of n-3 PUFAs negatively affects β-oxidation, meaning a reduction in liver lipid oxidation, which supports the idea of a link between a low dietary intake of PUFAs and NAFLD progression [48], whereas excess n-6 PUFAs are more related with steatohepatitis by induction of the intracellular oxidative stress, hepatocellular injury inflammation [49].

Besides, gene expression involved in adipose tissue lipid storage can be upregulated by microbiota, as demonstrated in different studies in animals and humans [50,51]. Therefore, the diet is an important exogenous factor, which directly contributes to the health state of the liver and the gut microbiota. Other key energy nutrients are sugars and an excessive energy consumption in the form of carbohydrates, especially fructose, which is a major risk of NAFLD development and severity of the disease [52]. Fructose is highly used in processed foods and beverages, and these products have usually been consumed in higher quantities by NAFLD patients, causing higher lipogenesis by upregulating the activity of the critical transcription factor, the sterol regulatory-element binding protein-1c (SREBP-1c), which promotes mitochondrial dysfunction [53]. Moreover, high amounts of fructose participate in the development of hepatic oxidative stress [54] and may also inhibit β-oxidation, which increases the amount of TG in the liver [55]. In addition, lower fiber, polyphenols, vitamins and mineral nutrients’ intake is associated with the development and progression of NAFLD [56,57].

Deficiency in both Vitamin D and copper increase the risk of inducing NAFLD [58,59]. Vitamin D inhibits liver fibrosis through the transforming growth factor beta (TGF-β) pathway and IR by the induction of the hepatic resistin [60]. In addition, deficiency of vitamin D stimulates the oxidative stress and inflammation of the liver via activation of some hepatic TLR receptors members [61]. On the other hand, deficiency in copper also promotes IR and hepatic steatosis, leading to lipid peroxidation [62]. Presence of high amounts of fructose also aggravates this situation, impairing copper absorption into the intestine, which contributes to enlarge liver damage [62]. Finally, polyphenols have been suggested as beneficial bioactive compounds for the prevention and the treatment of NAFLD. Notably, several studies have shown that polyphenols, including quercetin, epigallocatechin gallate, anthocyanins and resveratrol, can prevent the development of NAFLD by exerting lipid-lowering, antioxidant, anti-inflammatory and antifibrotic effects [63].

Taken together, it seems well established that diet may modulate NAFLD progression, and at the same time, diet is able to transform intestinal flora to a healthier or more harmful microbiota profile, making it necessary to deeply explore the straight connections between gut dysbiosis and non-alcoholic fatty liver disease.

## 4. How Variations in the Taxonomic Composition of the Gut Microbiota Affects NAFLD Progression

Several studies have obtained similar results regarding the differences at taxonomic level between the gut microbiota of healthy subjects and patients affected with NAFLD [29]. Usually theses variations might be beneficial for a resilience of the gut microbiota, but if a continuous external stimulation is stressful and disruptive, it might trigger unstructured microbiome, and dysbiosis contributes to NAFLD progression [29].

### 4.1. Changes at the Phylum Level

In the human gut, there are two dominant phyla of bacteria, *Bacteroidetes* and *Firmicutes*, which represent 90% of gut microbiota, and in less proportion, others phyla: Actinobacteria, Proteobacteria, *Fusobacteria*, and *Verrucomicrobia* [9,58]. Studies based on animal models have reported that HFD increases *Firmicutes* and *Proteobacteria* proportion, raising the ratio *Firmicutes:Bacteroidetes* [64]. On the contrary, human studies based in high-fat intake have demonstrated an opposite effect, with a decrease in *Firmicutes* and *Proteobacteria* proportion together with an increase of the *Bacteroidetes* [59]. In the same direction, a study comparing the gut microbiome between lean subjects and NAFLD patients, observed an increase of 20% in *Bacteroidetes* and a decrease of 24% in *Firmicutes*, with a higher gram-negative bacteria concentration, which showed an increase of the presence of pathogenic bacteria that produce LPS in NAFLD patients [20,63]. Reduction in *Firmicutes* is mainly a consequence of a decrease in SCFA-producing bacteria, such as the *Lachnospiraceae, Lactobacillaceae*, and *Ruminococcaceae* families [64]. Differences in the composition of the microbiota phylum between human and animals might be due to the differences in the type of fat that animals and humans consumed and absorbed [59]. Indeed, in animals’ models, it is described that an imbalance between the *Bacteroidetes* and *Firmicutes* ratio can alter mucin glycosylation [61]. In another human study, the abundance of *Firmicutes* and *Bacteroidetes* were similar between obese subjects and NASH patients, observing higher levels of *Proteobacteria* in NASH patients [42] and highly represented in fibrosis [62].

In one study it is described that ethanol-producing bacteria, from the phyla *Proteobacteria*, increased in metabolic abnormalities [65] and in NAFLD patients [66], contributing to intensify liver pathogenesis [66]. Moreover, it is reported that these heterolactic bacteria are involved in compromising the intestinal barrier integrity (leading a breakdown of the epithelial barrier), which compromise initiates mucosal inflammation and finally produces additional hepatoxical events [43], [66,67]. In addition, *Lentisphaerae*, a phylum with low representation in the gut microbiota, is observed being decreased in NAFLD patients in comparison with healthy patients [67]. Finally, *Fusobacteria* (a phylum with more bacterial pathogens, together with *Proteobacteria*) increased about 2.76% in NAFLD patients, causing the increase of the microbial gut toxins’ level [68].

### 4.2. Variations at the Family and Genus Level

Although there are many clinical and animal studies about changes at the family and the genus levels associated with NAFLD presence, those results are controversial [23]. In a mice model fed with HFD, whose members develop steatosis and metabolic disorders, gut microbiota was compared to the control-fed group, observing an increase of the *Barnesiella* and *Roseburia* genus and a decrease of the *Allobaculum* genus in the HFD group [23]. A clinical study compared the gut microbiome of 30 NAFLD patients *vs*. 30 healthy subjects and showed higher levels of those of the *Lactobacillaceae* and *Lachnospiraceae* families, but lower levels of the *Ruminococcaceae* family in NAFLD patients [68]. Furthermore, and in the genus level, were over-expressed the genera *Lactobacillus, Dorea, Robinsoniella* and *Roseburia*, but there was also under-represented the *Oscillibacter* in the same NAFLD patients [68].

These results were partially supported by a prospective cross-sectional study with 39 patients, being biopsy-proven of having NAFLD, observing that *Lactobacillaceae* family and *Lactobacillus* genus increased in the patients compared to healthy controls, whereas the *Coprococcus* and *Ruminococcus* genus levels decreased [69]. However, in obese children with NAFLD, there was observed an increase in the *Prevotellaceae* family because of the higher levels of the *Prevotella* genus [70].

Some studies found differences between healthy subjects and NAFLD patients and also between NAFLD early stages and NASH. One study viewed how the *Enterobacteriaceae* family and *Escherichia* genus were more present in NASH microbiota than in obese subjects, presenting a significantly elevated ethanol level in blood that probably contributed to the liver physiopathology [42]. A big difference found in a clinical study between NAFLD and NASH was the increased abundance of *Bacteroides* genus associated with the NASH presence [71]. *Bacteroides* caused an increase in the deoxycholic bile acid levels, which is related with the induction of apoptosis in hepatocytes [71]. These increases of the abundance of *Bacteroides* in NASH was inversely related to a decrease in *Prevotella* levels, contrary to the results obtained with NAFLD patients, in which this *Prevotella* level increased [71]. It might be a consistent finding due to the fact that *Bacteroides* and *Prevotella* are niche competitors, and the diet is a key contributor to influence in the levels of these genera in the gut: A Western diet is favorable for *Bacteroides*, whereas diets based on vegetables, fruits, plants polysaccharides and food rich in fiber are favorable for *Prevotella* [72]. Moreover, in the advanced stages of fibrosis, the genus increased was *Ruminococcus* [71], and in this genus there are species producers of alcohol, which could drive additional harmful actions on intestinal permeability and hepatic inflammation [72].

### 4.3. Variations of Specific Bacteria Associated with NAFLD

Several studies have enumerated the contribution in the NAFLD progression of specific gut microbiota bacteria affecting different processes: SCFAs homeostasis, de novo lipogenesis; VLDL metabolism; bile acid homeostasis; endogenous ethanol formation and increased levels of LPS, which is related to an inflammatory response in hepatocytes (Table 1) [73]. In this section, we will review recent studies that explain the contribution of some bacteria from the gut microbiota in the NAFLD physiopathology.

#### 4.3.1. *Faecalbacterium prausnitzii*

**Figure 1:**
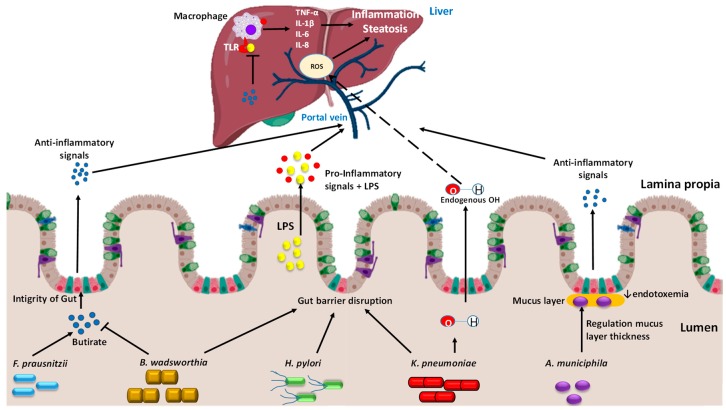
Pathways in the gut-liver axis of some bacteria which act differently in the gut and through the portal vein connected with the liver, contributing positively or negatively to NAFLD. In a healthy gut, *Faecalbacterium prausnitzii* contributes with the integrity of the gut, participating in the butyrate production, which interacts with the cells from the barrier modulating mucin and the tight junction’s formation, and the production of anti-inflammatory molecules. In dysbiosis, the microbiota concentration changes, and damaging bacteria grow above healthy bacteria. *Bilophila wadsworthia* reduces the production of secondary bile acids in the gut, while first bile acids are linked with the farnesoid X receptor (FXR) that provokes a decreased production of first bile acid in the liver, contributing to disrupted microbiota, and the increase of lipopolysaccharide (LPS) release. Moreover, it decreases the activation of butyrate production. *Helicobacter pylori* also participates in the gut barrier disruption, boosting the bacterial endotoxins’ passage to the liver, modulating pro-inflammatory cytokines and downregulating leptin and adiponectin. In dysbiosis, *Klebsiella pneumoniae* produces high quantities of endogenous alcohol, which arrives to the liver and increases the source of reactive oxygen species (ROS), related with NAFLD progression. *Akkermansia muciniphila* is found in the mucus layer of the gut barrier, which is reinforced due to the presence of *A. muciniphila* activity, modulating tight-junction proteins, regulation of mucus layer thickness and the promotion of antimicrobial peptides and immunity.

*Faecalbacterium prausnitzii* is a butyrate-producing bacterium, from the *Firmicutes* phylum (Figure 1) [84]. This bacterium is the dominant member of the *Clostridium leptum* subgroup, representing >5% of the total gut microbiota in healthy humans [84]. *F. prausnitzii* modulates the intestinal immune system, the oxidative stress and the metabolism of the colon epithelial cells (colonocytes) [85], and produces a microbial anti-inflammatory (MAM) protein [86]. In humans, it was observed that the low abundance of *F. prausnitzii* was associated with >5% fat hepatic content and with an increased adipose tissue inflammation, which may contribute to aggravate the NAFLD state [75]. These findings were corroborated in mice fed with HFD, observing that those animals that were treated with *F. prausnitzii* as a probiotic presented a lower hepatic lipid and lower plasma levels of liver transaminases (AST and ALT), suggesting a healthier liver than the one of the HFD counterpart mice (Table 1) [74]. Moreover, the presence of *F. prausnitzii* increases the expression levels of an inhibitor cell cycle progression, *CDKN1A*, which encodes for the p21 protein, whose levels are inversely associated with NAFLD progression and fibrosis [87].

#### 4.3.2. *Bilophila wadsworthia*

*B. wadsworthia* is a gram-negative *Proteobacterium* associated with fat rich diets [76]. This *B. wadsworthia* metabolizes sulfated compounds and produces hydrogen sulfide that promotes direct inflammation and impairs the gut barrier [76], and consequently an increased abundance of *B. wadsworthia* implies a negative effect in intestinal inflammation (Figure 1) [59]. In addition, a recent study demonstrated that hepatic lipid and triglyceride content increased in mice that had been fed with HFD and *B. wadsworthia* in comparison to HFD mice counterparts, which weakens liver function and potentiates metabolic syndrome [77]. As other gram-negative bacteria, *B. wadsworthia* may release LPS as endotoxin which stimulates a systemic inflammatory response, raising the circulating levels of key cytokines such as serum amyloid A (SAA) and interleukin-6 (IL-6) [76]. Besides, *B. wadsworthia* decreased butyrate metabolism, which interrupts the tight junction integrity of the gut barrier, allowing the circulation of LPS from the gut lumen into the portal vein arriving to the liver, where it acts upon hepatic macrophages, increasing a pro-inflammatory cytokine release (Table 1) [77]. Finally, *B. wadsworthia* promotes a reduction of the primary bile acids’ production, contributing to a disrupted microbiota, and the increase of LPSs release [82,83].

#### 4.3.3. *Helicobacter pylori*

*Helicobacter pylori* is a gram-negative *Proteobacterium* and represents, in humans, a key factor in the etiology of various gastrointestinal infections [88]. Several studies have observed a significantly increased risk of NAFLD in patients affected by an *H. pylori* infection, and this bacterium also plays an important role in IR, which is described as a factor of NAFLD’s development due its chronic inflammation state [85,89]. When the infection is eradicated, the risk of NAFLD development is reduced. Besides, *H. pylori* infection modulates the release of several inflammatory cytokines (tumor necrosis factor α (TNF-α) and some interleukins, IL-1β, IL-6 and IL-8) which drive an important role in hepatocellular injury associated with NAFLD (Figure 1) [80]. In addition, leptin release from white adipose tissue is induced by *H. pylori* infection [80]. Leptin is a key adipokine which contributes to IR by its role in the regulation of glucose, energy homeostasis and lipid metabolism. Increased leptin levels activate the liver stearoyl CoA desaturase, thus accelerating VLDL formation and fat deposition in the liver [80]. Finally, *H. pylori* infection has the greatest impact on the homeostasis of upper digestive tract, which affects the gut–liver axis. This bacterium might increase the mucosal permeability of the gut and cause flora dysbiosis, thereby boosting the bacterial endotoxins passage to the liver through the portal vein circulation [80]. These endotoxins trigger pro-inflammatory cytokines release such as TNF-α and interleukin-8 (IL-8) via TLR, which triggers the hepatic migration of neutrophils and monocytes [89], increasing IR and lipid accumulation in the liver (Table 1). However, more clinical studies are necessary to understand the complete role of *H. pylori* in the NAFLD progression.

#### 4.3.4. *Klebsiella pneumoniae*

In a healthy state, microbiota is constantly producing ethylic alcohol in the gut, which is normally metabolized in the liver by alcohol-dehydrogenase (ADH) and other hepatic enzymes [90]. When the gut microbiota is enriched in alcohol-producing bacteria, the production of alcohol is more constant than in healthy microbiota, exceeding the liver detoxification capacity and therefore producing a constant source of reactive oxygen species (ROS) towards the liver, which induce hepatic inflammation, often ending in steatohepatitis [90,91]. In fact, more bacterial species with stronger alcohol-production ability have been shown in patients with NAFLD than in control patients [81,92]. In a very recent study, two strains of a same bacterium were identified, which are related with endogenous alcohol production and is more abundant in NAFLD patients, *K. pneumonia,* a gram-negative *Proteobacterium* [81], a phylum significantly elevated in NASH [92].

Moreover, both in aerobic and anaerobic conditions, *K. pneumoniae* induced a higher blood alcohol concentration (BAC) in NAFLD patients than in control patients due to its higher alcohol-producing ability [81]. In addition, after inducing a reduction in the *K. pneumoniae* abundance, a double effect was observed: A body weight loss and a decreased endogenous alcohol production by the fecal flora, suggesting an association between *K. pneumoniae* presence and NAFLD progression (Figure 1) [81]. In mice, the transplant of *K. pneumoniae* is sufficient to induce hepatic NAFLD, increasing triglycerides, ALT and AST concentration in the serum [81]. Different pathways were also affected, with increased expression of genes related to progressive fat storage, enrichment of the biosynthesis of unsaturated fatty acid and other metabolisms related with the development of hepatic steatosis and inflammation [81]. In fact, in NAFLD mice induced by *K. pneumoniae;* higher alcohol concentrations are reported in the portal vein than in the peripheral veins, demonstrating the alcohol production by the microbiome [81]. Moreover, these evidences were corroborated in NAFLD patients, because endogenous alcohol production is enlarged, possibly due to the actions of *K. pneumoniae* (Table 1) [81]. To sum up, *K. pneumoniae* contributes to the NAFLD physiopathology with an etiological behavior similar to alcoholic fatty liver disease.

#### 4.3.5. *Akkermansia muciniphila*

*Akkermansia muciniphila*, a gram-negative bacterium from the *Verrucomicrobia* phylum, is one of the most abundant microorganisms in the human intestinal microbiota, representing between 3–5% of the whole bacteria community [82]. It is described as a beneficial microbe, which could be considered as a potential probiotic treatment [93]. This bacterium is found in the mucus layer of the intestine, with a mucin-degrading activity [94], and it is established in the intestine during the first month of life [83]. In a mice model, *A. muciniphila* is less abundant in obese and NAFLD animals than in their counterparts [93]. This decrease is also inversely correlated with fat mass gain, body weight, inflammation, IR and glucose tolerance [93]. Besides, a thinner intestinal mucus layer was observed in obese animals, causing greater gut permeability and allowing the entrance of bacterial compounds into the circulatory system [94]. Accordingly, a higher presence of *A. muciniphila* induce an improvement in metabolic disorders, lowering the cholesterol levels and liver steatosis (Table 1) [93]. It has been shown that metformin, widely used as a first-line antidiabetic treatment, improved glucose homeostasis correlated with an *A. muciniphila* increased population [93]. Reinforcement of gut barrier and the reversed fat gain has been related to the increase of the circulating levels of endocannabinoids and gut peptides, due to the presence of *A. muciniphila* activity, modulating tight-junction proteins, regulation of mucus layer thickness and the promotion of antimicrobial peptides and immunity [93].

*A. muciniphila* is capable of obtain carbon, energy and nitrogen source from mucin and then releases free sulfate from mucin fermentation [94]. Amuc_100 is described as a protein synthetized by the *A. muciniphila* layer that plays an important immunomodulatory role [94]. A study from S. Zhao et al. in preclinical models of specific pathogen-free (SPF)-grade mice described the improvements in metabolic profiles due to daily supplementation by gavage of *A. muciniphila* [83]. The findings resulted in an improvement of glucose tolerance and insulin sensitivity in the liver by a significant reduction in the expression levels of genes involved in the glucose metabolism (phosphoenolpyruvate carboxykinase (PEPCK) and glucose-6-phosphatase (G6PC)) [83]. Besides, a significant reduction in the expression levels of liver genes involved in fatty acid synthesis (SREBP1c) and transport (fatty acid translocase (CD36)) were observed due to *A. muciniphila* supplementation, which drives to a lower fat deposition and ER stress induced by *A. muciniphila* in this essential organ [83]. In addition, the plasma lipopolysaccharide binding protein (LBP) binds LPS, helping LPS to be recognized by the TLR4 receptor, initiating downstream signaling that results in inflammation [83]. LBP levels were reduced in systemic circulation by the increased presence of *A. muciniphila*, reducing metabolic endotoxemia and downstream signaling [83]. Taken together, these metabolic benefits induced by *A. muciniphila* could provide possibilities to prevent or ameliorate health disturbances in the general population.

## 5. Future Perspectives

Apart from the extended studies focused on bacterial gut microbiota, intestinal flora are also composed by nonbacterial members that may develop an important role in the processes affecting health and disease. Eukaryotes contribute to less than 0.03% of the total fecal microbes and are primarily composed of 200–300 fungal species [95]. Fungi have been found altered in the gut microbiome by some diseases that affect gut permeability, such as inflammatory bowel disease (IBD) [96]. In fact, obese mice fed with kefir, which contains yeast as *Saccharomides spp* and *Candida spp*, ameliorate NAFLD, with improvements in hepatic lesions and lower levels of steatosis [97]. These results point at yeast as an important contributor in the improvement of NAFLD progression and its necessity to have yeasts in mind in order to design effective strategies against the development of NAFLD. Other nonbacterial members of the gut microbiota are virus and a meta-analysis of gut virome, which have displayed that bacteriophages compromise the 90% approximately of the gut virome and have an important participation in bacterial dynamics and mechanisms in gut microbiota [98]. Despite the important role of virus over the gut homeostasis, the potential effects of gut virus in the NAFLD development has not been explored yet, making it necessary to consider this virus as an additional tool to fight against this important disease in a holistic manner.

In addition, both the gut microbiome and NAFLD are closely connected with the circadian clock. Microbiota rhythms are regulated by diet and time of feeding which can alter both microbial community structure and metabolic activity that can significantly impact metabolic function [99]. Indeed, an increasing number of circadian rhythm studies have provided important insights correlating the expression of the circadian clock gene with metabolism in NAFLD [100]. However, the exact mechanisms of circadian metabolism remain obscure and unresolved at this time, and clearly require additional experimentation to further increase our comprehension of lipid metabolism in the liver. Therefore, new studies that target key circadian clock genes with the aim of treating or preventing NAFLD may provide more effective strategies of intervention in the future.

## 6. Conclusions

To sum up, gut microbiota plays a significant role in the pathogenesis of obesity and NAFLD progression [47]. Gut dysbiosis and bacterial translocation in combination with a Western diet and lifestyle with inflammasome dysfunction lead to NAFLD progression [62]. This dysbiosis produces an increase in harmful bacteria and/or a decrease in beneficial bacteria, affecting the health of both the intestine and the liver. The mechanisms modulated by these bacteria should be further investigated to know where and how they affect the principal pathways, which involve the homeostasis of the intestine and the liver health. Moreover, the mechanisms that clarify the link between ingredients and metabolites from the gut microbiome with NAFLD have been analyzed in some studies, but it is necessary to devote more efforts in this field to obtain a complete picture. This review summarizes the assessed and characterized bacteria related with the NAFLD progression in depth. However, considering the high number of different species that are present in the gut microbiota, it is logical to think that other bacteria and non-bacteria species may play an important unknown role in the development of this disease.

## Figures and Tables

**Table 1:** Summary of bacteria that are directly related to the progression of Non-Alcoholic Fatty Liver Disease (NAFLD).

Bacteria Species	Characteristics	Main Effects	Experimental Models	Refs.
***Faecalibacterium prausnitzii***	*Firmicutes* phylum. Butyrate-producing bacteria. >5% of the total gut microbiota in healthy humans.	↓ [*F. prausnitzii*] → >5% fat hepatic content and ↑ adipose tissue inflammation in humans. ↑ [*F. prausnitzii*] → Lower lipid content, ↓ ALT and ↓ AST, ↑ *CDKN1A* (inversely correlated with NAFLD), ↓ hepatic fibrosis.	Clinical study with 31 participants with high hepatic fat content. HFD mice fed with *F. prausnitzii* vs. control mice and HFD mice not fed.	[74,75]
***Bilophila wadsworthia***	Gram-negativeBacteria. *Proteobacteria* phylum. Associated with diets rich in fat.	↑ [*Bilophila wadsworthia*] → ↑ Serum liver enzymes, hepatic steatosis and ↑ cholesterol levels. ↑ Hepatic lipid content and ↑ hepatic TG, ↓ butyrate metabolism pathway activation and ↑ SAA and IL-6. ↑ [*Bilophila wadsworthia*] → Stimulate systemic inflammation, intestinal barrier defect, bile acid dysmetabolism and interrupting tight junction integrity.	*B. wadsworthia* infection on specific pathogen-free mice. HFD mice fed with *B. wadsworthia.*	[76,77]
***Helicobacter pylori***	Gram-negative Bacteria. *Proteobacteria* phylum. Common infection in humans.	↑ [*H. pylori*] → -↑ Chronic inflammation and IR → ↑ TNF-α, IL-1β, IL-6 and IL-8. ↑ [*H. pylori*] → ↓adiponectin and leptin, ↑ fetuin-A → cytokines release ↑ [*H. pylori*] → ↑ Flora dysbiosis and mucosal permeability → ↑endotoxemia.	Clinical studies of NAFLD patients with *H. pylori* in a meta-analysis study.	[78,79,80]
***Klebsiella pneumoniae***	Gram-negative Bacteria. *Proteobacteria* phylum Endogenous alcohol-producing bacteria.	↑ [K*. pneumoniae*] → ↑ Endogenous OH → ↑ ROS, ↑ hepatic steatosis, ↑ TG, AST and AST. ↑ Immune cells and inflammation in liver ↑ Biosynthesis FAs and Fat storage	Clinical study with NAFLD and controls individuals colonized by *K. pneumoniae*. NAFLD mice induced by *K. pneumoniae.*	[81]
***Akkermansia muciniphila***	Gram-negative Bacteria *Verrucomicrobia* phylum 3–5% of the Gut microbiota Mucin-degrading activity.	↓ [*A. muciniphila*] → ↑ obesity, metabolic disorders, ↑ fat mass gain, ↑ body weight, ↑ inflammation, ↑ insulin resistance and ↑ glucose tolerance ↑ [*A. muciniphila*] → ↓ metabolic disorders, ↓ obesity, ↓ insulin sensitivity, ↓ fat mass and liver steatosis, ↓ [cholesterol] Regulation of mucus layer thickness → ↓ permeability → ↓ Endotoxemia	*A. muciniphila* in HFD-induced obese mice *A. muciniphila* supplementation in specific pathogen-free-grade mice.	[82,83]
